# Understanding Patient Values and the Manifestations in Clinical Research with Traditional Chinese Medicine—With Practical Suggestions for Trial Design and Implementation

**DOI:** 10.1155/2013/847273

**Published:** 2013-12-02

**Authors:** Wei Mu, Hongcai Shang

**Affiliations:** ^1^Center for Evidence-Based Medicine, Tianjin University of Traditional Chinese Medicine, 312 Anshanxi Road, Nankai District, Tianjin 300193, China; ^2^Tianjin Institute for Clinical Evaluation, Tianjin University of Traditional Chinese Medicine, 88 Yuquan Road, Nankai District, Tianjin 300193, China

## Abstract

*Objective*. To define patient values, identify their manifestations in a randomized clinical trial, and investigate the possible implications for clinical research of traditional Chinese medicine. *Methods*. We categorized patient values manifestations into patient choice, preference, compliance, and patient-reported outcomes and summarized the underlying personal values through purposeful electronic searches for relevant reports. By hypothesizing a set of positive versus negative circumstances occurring in the enrollment, intervention allocation, treatment, and the follow-up stage of a trial, it is possible to discuss the potential implications of patient values manifestation on a trial with traditional Chinese medicine. *Results*. Patient values and its manifestations are ubiquitous in the process of clinical research with traditional Chinese medicine. These values may provide motivation for participation or engender the internal and external validity of the study. *Conclusions*. Trialists should attach sufficient importance to the needs and concerns of individual participant. To incorporate patient values into the design and conduct of a clinical study with traditional Chinese medicine, researchers are recommended to adopt participant-friendly design and use patient-reported outcomes, take convenience-for-patients measures, and help foster rational beliefs and behaviors of trial participants.

## 1. Introduction

Patient values are depicted as the unique preferences, concerns, and expectations each patient brings to a clinical encounter in the EBM sense [1]. Personal and cultural values reflect an individual's sense of right and wrong or what one ought to do, and tend to influence attitude and behavior. In the context of clinical trials where patient engagement is most intensive, values have a wider meaning and a larger role to play. In this study, the authors defined patient values as a set of personal codes of conduct by which a patient judges what is important, makes decisions, and takes actions. Deeply grounded in a person's experiences, cultural background, economic circumstance, and religious beliefs, patient values are empirical and subjective in nature, yet susceptible to suggestions from the doctor and one's relatives and friends as well as from other factors.

In the clinical research of traditional Chinese medicine (TCM), the randomized controlled trial (RCT) design is generally acknowledged as the gold standard for efficacy assessment. It uses a prospective experimental design with unique features that enable the investigator to proactively control intervention assignment and other confounders that may inflict on the validity of the results. Properly conducted randomization can help eliminate bias in treatment assignment, facilitate the masking of treatment group, and maximize statistical power in terms of introducing equal group sizes [2].

Despite the “standard patient” and the “standard treatment procedure” claimed by pro-RCT methodologists, the individuality of trial participants and, especially for TCM clinical research, the interactive and patient-centered nature of TCM interventions are never to be undermined. While study subjects are expected to be actively involved in the whole process of the trial, one can hardly tell why eligible candidates are unwilling to enroll, or what makes enrolled subjects refuse randomization, fail to keep a schedule, or discontinue treatment, and after all how the participants' values take a role in this. These problems need to be carefully discussed and the participants' needs and concerns need to be well incorporated into trial design and implementation to avoid detrimental effects on the trial results.

## 2. Materials and Methods

A number of cross-sectional surveys and interview studies [3–6] have examined similar questions, but a general picture depicting the scale of the impact of patient values on clinical research in TCM has not been fully unfolded. In this report, a preliminary attempt was made to summarize patient values and its manifestations in a hypothesized set of positive versus negative circumstances in the context of the enrollment, randomization, intervention allocation, treatment duration, and follow-up phase of a RCT. Patient values and beliefs that may impact their behavior in a clinical trial were identified through purposeful rather than comprehensive electronic searches. The finding from systematic reviews was quoted in preference to that of a survey. The RCT model was chosen because the design widely acknowledged the most rigorous way of removing extraneous factors and any potential confounding variables, but in this study we will show you why the RCTs cannot avoid influences from patient values. We categorized values' manifestations into patient choices, patient preferences, compliance, and patient-reported outcomes and provided a definition for each, identified the underpinning personal values, elaborated on possible implications, and came up with advices on study design and implementation.

## 3. Results

### 3.1. Patient Choices

Having choices and making decisions are parts of the life of patients involved in a RCT. Here, we restricted the scope of the term to refer to the act of a candidate subject choosing whether or not to enroll in the study, or to readily accept the allocated medication. Personal values cannot only influence a candidate subject's decision-making as to whether to participate by giving informed consent, and go on to impact on the enrolled participant's willingness to continue after being informed of the allocated group as in an unblinded study. For instance, patients who believe that “human energy (*qi* in Chinese *pinyin*) is consumed during acupuncture” may never volunteer for a RCT with acupuncture. Even if they do get involved, it is very likely that they will drop out of the study once they are informed about being assigned to the acupuncture group. In the case of an unsuccessful recruitment where a low proportion of eligible candidates consent or remain in the study after disclosure of allocation, the progress of clinical research will be delayed and excessive costs incurred, and the study may risk early termination.

According to two systematic reviews [3, 4] on patient-reported barriers to participation in RCTs, main causes of concern that are generally value-based involve participants' belief that they should be paid, their questioning of the treatment efficacy, a distrust of hospital or medication, and the fear for the unknown. Despite repeated searches, we failed to locate reports on patient values-based reasons for refusal of participation in the TCM clinical research. However, there is indirect evidence from a survey of 2,000 potential trial candidates in a hospital in west China which identified factors associated with the Chinese patients' unwillingness to participate in clinical research [7]. Many of them relate to personal values, which are the fear of being a “guinea pig” in the trial setting, concerns with drug safety, efficacy and side effects, and the belief that a trial participant will be marginalized as “someone special.” While these factors have their roots primarily in the participant's self-belief systems, influences from the outside world such as the oppositions from an “important person” (e.g., spouse, parent, close friend) and the attitudes of their clinicians have also been reported to be capable of reshaping patients' beliefs and facilitating decision-making [4].

Therefore, it is advisable to take advantage of external influences such as education programs, consultancy services and in-depth physician-patient communication to inform knowledge, increase understanding, and resolve doubts. In clinical research of TCM, especially the potential benefits and risks of all treatment options, shall be explained in ways that the participants accept and understand, rather than using obscure TCM terminology and philosophical reasoning. Potential participants should be informed of what clinical research is, what it does, and their rights and obligations, and most importantly they are cleared of all their misunderstandings of clinical research and of the TCM therapy to be applied in pretrial consultations. Furthermore, a good doctor-patient relationship also has a role to play in enhancing the rate of successful referrals for a trial.

### 3.2. Patient Preferences

Patient preferences denote a patient's expressed greater interest in or desire for a treatment option than any other ones. After randomization and in intervention allocation, patients' personal values manifest themselves by making choices regarding whether or not to have the assigned medication (no matter one desired it or disliked it), on the condition that patients are aware of the treatment to be administered (such as in a design where masking of patients is not used). This suggests that unblinded studies are particularly vulnerable to possible patient preferences-induced biases.

Unfortunately, the development of methodology for trials with TCM has not been sufficiently sound to provide appropriate placebo designs for many TCM therapeutic tools (acupuncture, cupping, etc.). Therefore, the use of blinding is impractical. In placebo-controlled trials with herbal medicine or Chinese patent drug, similarly, the placebo may not be perfectly indistinguishable from real drug because the taste, color, and smell of a herbal decoction are absolutely unique. A proportion of participants may know of the allocation during the trial, especially when they have previously taken the drug of interest. As a result, a major portion of clinical studies in TCM were unblinded. In proof of this statement, we launched a search on the Cochrane Central Register for Controlled Trials (Issue 8, 2013) to identify the number of controlled clinical trials with TCM and the ratio of blinded versus unblinded studies. It was found that only 129 among the total of 628 controlled trials used blinding. In these unblinded trials where the participants are aware of the treatment, preferences for one treatment option may influence their willingness to continue treatment, their expectations, if any, and the degree of engagement in the study. For example, patients preferring one kind of Chinese herbal medicine to the other in a trial might respond negatively to being randomized to the unopted arm.

This could lead to two possible circumstances. The first one is increased drop-outs and reduced statistical power. Findings from one systematic review [8] showed that patient preferences for a treatment option could prevent them from attending the trial or accepting the treatment assigned. If patients with strong preferences for one treatment regimen are not assigned to their opted arm, and thereby decide that they will not proceed further in the study, the implications for the trial would be much more serious than when the same withdrawals occur before randomization because this may lead to reduced comparability between groups right at the start [9]. The absence of these participants (eligible but withdraw for preferences) may restrict generalization of the results and weaken the external validity of the study [8, 10].

Secondly, if patients with strong preferences remain in the trial after being randomized to the nonopted intervention, they may fail to adhere to or passively receive the assigned treatment or even seek the other treatment option on their own [11, 12]. This leads to implications for compliance, and the contamination of interventions. On the other hand, those allocated to their preferred treatment may expect higher of the therapy, engage more actively, and believe firmer in the treatment efficacy, which all might contribute to better clinical outcomes by exerting positive psychological effects similar to the placebo effects [13]. The phenomenon has been evidenced by the findings of a systematic review and patient level meta-analysis of 17 musculoskeletal trials, which found patients randomized to their preferred treatment did better than participants who were indifferent to allocation or those who received unopted treatment [14]. However, other studies [8, 15] came to contradictory results and found insufficient empirical evidence in proof of consistent effect of preference on outcomes.

To incorporate patient preference into the design of clinical trials, preference controlled designs such as the comprehensive cohort design, the Rücker design, and the Wennberg et al. design have been proposed as alternatives to the conventional RCTs. In the comprehensive cohort setting, participants with preferences are allowed their desired treatment and those with no preferences are randomized as usual [16]. The Rücker's design randomizes half of the study sample to a choice group and the other half to a randomized group. Only participants in the choice group have the chance to choose their desired treatment or to be randomized [17]. In the Wennberg et al. design, participants are randomized to a preference group, in which all patients decide for themselves the treatment to receive, or to a randomized group.

Back to clinical trials with TCM, apart from the above mentioned difficulties in blinding, for which the patients are either aware of treatment allocation or it is easy for them to detect the allocation, the efficacy evaluation system of TCM also relies heavily on self-reported outcome measures (i.e., changes in TCM symptom scores), which are considered sensitive to personal preferences. These features are in alarming resemblance to the conditions, what Halpern [18] described as the seedbed for preference-induced biases. Furthermore, trials with nonpharmacological interventions such as acupuncture, moxibustion, and cupping involve lots of participant engagement, corresponding to what Brewin and Bradley [19] termed as the “participative interventions,” in which preference effects are likely to be most apparent. In view of this, the preference controlled design provides an attractive alternative for clinical research of TCM despite the fact that it may require a larger sample, take longer, and cost more.

### 3.3. Patient Compliance

Compliance is narrowly defined in this paper as the extent to which a study subject follows the treatment regimen as required, in terms of taking medications, following diets, executing lifestyle changes, or paying visits [5], during treatment and followup. In trials with TCM treatment regimens where patient motivation and engagement play a key role, compliance or noncompliance behaviors on the part of the participants will make a big difference in the trial results. Both differential and similar dropout rates across trial arms may engender the comparability of groups, and high attrition rate also limits the interpretation of trial results [20].

Factors relating to patient compliance are multifaceted, but sociopyschological studies have suggested that values underpin patient behaviors in a clinical study. When it comes to what kind of values turn out to compliance and what to poor compliance, a review [6] of factors from the patient's perspective gave us some hints.

In summary, good compliance has been found to be connected with the following patient values:

- feeling susceptible to a disease or its complications;
- believing the disease or complications may end up with severe consequences;
- believing in the efficacy or benefits of the treatment.

More items of values have been reported to relate to poor compliance behaviors, including the following:

- believing that long-term use of western medicine was harmful;
- worrying about diminished effectiveness of medication over time;
- fearing to develop dependence on long-term use of drug;
- perceiving less need for drug as disease is God's will and is uncontrollable;
- having low motivation to change behaviors or take medication;
- having negative attitudes or even depression;
- feeling stigmatized while on medication.

Some of the above values apply well to patients attending clinical trials with TCM. Generally speaking, TCM regimens applied in clinical research feature a long duration of treatment, slow onset of action, and enduring course of effects. In trials testing Chinese patent medicine for chronic stable coronary artery disease, for example, the participants may be highly motivated at the beginning, but their patience could be toiled by the long treatment duration (usually lasting for months in addition to a 1-year follow-up period), and they may feel disappointed if the treatment effects are less prominent than expected. They may also feel good about their health after weeks of treatment and begin to take fewer drugs or even miss treatment sessions by their own free will. Along with disappointment, fulfillment of expectations, and other negative or positive experiences with the therapy in a TCM trial, a variety of new beliefs and personal codes of conduct might be formed. Many of these could lead to good or poor compliance. However, patients' psychological changes predictive of nonadherence are subtle and hardly perceptible, thus, posing challenges to the trialists.

Many methods and techniques have been developed to monitor and manage the noncompliance phenomenon, but few of them were based on the patients' needs and values. Here, we propose the use of in-depth physician-doctor communication during or after each treatment session (e.g., during acupuncture sessions) to detect possible predicators of any non-compliance behavior so that the trialists can take measures accordingly. For instance, the participants could be clearly informed of the potential health hazards that resulted from irregular or discontinued treatment and of the benefits of adherence to the treatment regimen. More physician attention, and social support, perhaps also financial help should be extended to all participants to prevent them from feeling depressed, isolated, or utilized. This approach could also be part of the qualitative research projects nested within TCM clinical trials for deepening understanding on patient experiences with complex interventions, as proposed by Liu [21].

Empirical evidence from clinical research in TCM showed that an emphasis on the improvement of patient-important outcomes, rather than laboratory indicators, could be more attractive to the patients and therefore such study design could have a better profile of participant retention. In a randomized trial testing the superiority of alendronate sodium tablets plus acupuncture over alendronate sodium tablets for osteoporosis, the former group had a dropout rate of 72% compared with 32% of the latter group over a 12-month treatment period, and with statistically significant differences (*P* < 0.05) [22]. The main reason is that the acupuncture sessions provided extra pain relief for patients in the treatment arm (reduction in VAS scores, *P* < 0.05). And for patients suffering from osteoporosis, alleviation of pain counts a lot.

### 3.4. Patient-Reported Outcomes

The patient-reported outcome (PRO) is an umbrella term for all patient assessments of a health condition and its treatment [23]. By definition, it relies heavily on the subjective perceptions of the patient himself and therefore captures well his experiences and perspectives [23], reveals what matters and what does not to him [24], and constitutes part of the representations of personal values. During data collection (in the treatment duration or during followups), patients might be required to complete items in a PRO instrument, describing changes of symptoms, health functioning, and sense of wellbeing or reporting on the perceived efficacy, safety, and acceptability of the treatment. Process evaluation such as patient reported satisfaction and compliance is another dimension of the PRO instrument. While medical studies increasingly crave the humanistic feature, PROs are winning places in both clinical trials and drug approvals in the TCM field.

A successful PRO instrument is the product of systematic item collection, rigorous psychometrics verification, and revisions from repeated pilot surveys, which satisfies certain development, psychometric and scaling standards. A PRO tool for TCM also incorporates many aspects that are characteristics of the Chinese medicine, such as the introduction of measurement items relating to the human constitution, the unity of physical and mental health, and the balance between the person and the nature. Moreover, items concerning signs and symptoms relevant to TCM pattern diagnosis are given prominence in a PRO instrument for TCM, such as the sense of taste, sleep pattern, energy and sound, and status of urine and stool. Most importantly, the construction of such a PRO follows a conceptual framework guided by the TCM theories [25]. As Liu noted, “the application of PRO instruments in TCM could be a large step towards the scientific and standardized efficacy evaluation of TCM” [26].

## 4. Discussion

It seems that patients have values and the manifestations of these values tend to be ubiquitous in clinical trials with TCM (see Table 1 for a summary of these manifestations). They either turn out to behaviors that impede the progress of a RCT or those that facilitate it. To manage these desirable and undesirable humanistic features of clinical studies, patient-friendly trial design and convenience-for-patients measures are recommended. Preference controlled trials that allow the participant to choose from available treatment options of one's own free will, the introduction of patient-reported outcomes that matter to the participants, and other innovative measures fostering convenience of participation could provide valuable references for future trialists. Furthermore, there is more we can do to improve the quality of clinical research in TCM now that we know what kind of patient values may pose what type of risk of bias in every stage of the trial. Initiatives shall be taken to weaken negative influences by guiding patient values. It is time to confront the challenge and do something.

## 5. Conclusions

Patient values and the various manifestations tend to have wide implications for clinical research in TCM. It is recommended that trialists respect these values, use participant-friendly design and patient-reported outcomes, take convenience-for-patients measures, and help foster rational beliefs and behaviors of the participants in future clinical trials with TCM.

## Figures and Tables

**Table 1:** Manifestations of patient values in each phase of a randomized controlled trial.

Phase	Circumstance A	Circumstance B	B's implications	Patient values' role	Manifestations
Enrollment	Gave informed consent including consent to randomization	Refused to give informed consent and did not admit	Delays of recruitment Reduced sample size	Made judgments	Choices
Randomization and intervention allocation	Agreed to receive allocated treatment	Randomized to an unopted treatment and refused to continue	Ended participation Decreased statistical power	Made judgments	Choices
		Attended preference controlled trials	Entered the opted group	Directed preferences	Preferences
Treatment duration	Received treatment as required	Received treatment not as required	Violation of protocols	Guided behaviors	Compliance
		Withdrew from treatment or participation	Withdrawals		
	Reported outcomes			Made judgments	Patient reported outcomes
Followup	Completed all visits as required	Missed visits	Violation of protocols	Guided behaviors	Compliance
		Withdrew from the trial	Lost to followup		
	Reported outcomes			Made judgments	Patient reported outcomes
